# The effects of grammatical gender on the processing of occupational role names in Slovene: An event-related potential study

**DOI:** 10.3389/fpsyg.2022.1010708

**Published:** 2022-12-15

**Authors:** Jasna Mikić Ljubi, Andraž Matkovič, Jurij Bon, Aleksandra Kanjuo Mrčela

**Affiliations:** ^1^Center for Organizational and Human Resources Research, Faculty of Social Sciences, University of Ljubljana, Ljubljana, Slovenia; ^2^Department of Psychology, Faculty of Arts, University of Ljubljana, Ljubljana, Slovenia; ^3^University Psychiatric Clinic Ljubljana, Ljubljana, Slovenia; ^4^Department of Neurology, University Medical Center Ljubljana, Ljubljana, Slovenia; ^5^Department of Psychiatry, Faculty of Medicine, University of Ljubljana, Ljubljana, Slovenia

**Keywords:** N400, P600, match-mismatch paradigm, grammatical gender, Slovene language, event-related potential (ERP)

## Abstract

The event-related potential method has proven to be a useful tool for studying the effects of gender information in language. Studies have shown that mismatch between the antecedent and the following referent triggers two ERP components, N400 and P600. In the present study, we investigated how grammatical gender affects the mental representation of the grammatical subject. A match-mismatch paradigm was used to investigate how masculine grammatical gender and gender-balanced forms (the explicit mention of masculine and feminine forms as word pairs) as role nouns affect the processing of the referent in Slovenian. The morphological complexity of Slovenian language required the use of anaphoric verbs instead of nouns/pronouns, on which previous research was based. The results showed that following both the gender-balanced and the masculine generic forms, P600 (but not N400) was observed in response to the feminine verb but not to the masculine verb. The P600 amplitude was smaller in the case of the gender-balanced form than in the case of the masculine generic form only. We have concluded that gender-balanced forms are more open to feminine continuations than masculine generic forms. This is the first ERP study in Slovenian to address the effects of processing grammatical gender, thus contributing to existing research on languages with grammatical gender. The great strength of the study is that it is one of the first ERP studies to test the mental inclusivity of gender-balanced forms.

## Introduction

Gender occurs universally in language and is automatically activated/processed in every social situation (Fiske, 1998; Fiske et al., 1999). There are several different ways of expressing gender in language. On the one hand, there are languages in which gender has almost no grammatical manifestation, i.e., so-called genderless languages (e.g., Turkish, Hungarian, Finnish, Chinese languages) (Stahlberg et al., 2007). We also know of languages with conceptual or semantic gender (e.g., English, Scandinavian languages), which are characterized by the absence of gender labels because most personal nouns are not gendered and can therefore be used to address women and men alike. In these languages, inferring the gender of a person explicitly named (e.g., James, Rachel) or situations where gender is expressed in kinship terms (sister, brother) is a straightforward and usually obvious process. However, when the person is referred to by a role name (e.g., doctor), the process becomes more complex (Gygax and Gabriel, 2011) because role names are not explicitly marked with gender (Irmen, 2007). Several cognitive processes are usually involved in the processing of role names. An important feature of role names in these languages is that they are particularly susceptible to gender stereotypical information (Baudino, 2001), which is why they are often used in studies examining the influence of gender stereotypes on reading comprehension (see, e.g., Garnham et al., 2002; Kennison and Trofe, 2003; Sturt, 2003; Duffy and Keir, 2004).

On the other hand, in languages with grammatical gender (e.g., Germanic, Romance, and Slavic languages–Slovenian), it is impossible not to mention gender when we talk about people since each noun is ascribed a gender (feminine or masculine). Feminine and masculine forms are usually distinguished by their suffixes; however, gender markers are also found in articles, adjectives, verbs, or pronouns (Braun et al., 2005; Stahlberg et al., 2007). In languages with grammatical gender, the mental representation of gender usually depends on grammatical gender (Gygax et al., 2008; Garnham et al., 2012). Thus, role name processing depends on gender agreement rules (and their violation) (e.g., Deutsch and Bentin, 2001; Schmitt et al., 2002), but in some cases also on the prevailing semantic characteristics of role names. Difficulties in forming clear mental representations usually occur when a role noun is presented in the masculine form. The masculine plural form can be interpreted either as a specific form referring to a group of men or as a generic form referring to both women and men. Therefore, there is always gender ambiguity when using this form.

In neurolinguistic research, the effects of grammatical gender and its semantic characteristics are usually determined by the resolution of anaphora in reading. An anaphora is used as a grammatical substitute (such as a noun, pronoun, or verb) to denote a word or group of words that occurs at the beginning of a sentence (also called an antecedent) (Merriam-Webster, 2020). The resolution of anaphora depends largely on the match/agreement between the grammatical and/or semantic characteristics of the antecedent and the anaphora. For example, a noun may be grammatically masculine, feminine, or neuter, but it can also be semantically associated with notions of masculinity or femininity–both aspects may influence our interpretation of the anaphora (Garnham, 2001). Gender-agreement rules are part of syntax theory (Haegeman, 1991), which ensures that words and phrases are logically combined in a sentence; that is, syntax rules determine the order of sentence elements and their grammatical role (Osterhout and Holcomb, 1992).

Based on interlingual comparisons of different languages that were comparable in their stereotypes but differed in their grammatical structure, studies (Garnham et al., 2002; Irmen and Roßberg, 2004; Gygax et al., 2008) suggest that grammatical gender can prevail over semantic (stereotype) information in language. For example, the word *les infirmiers* (the nurses) most likely evokes the representation of women in French. However, when written in the masculine form, it can lead to a contradiction between the grammatical form and the stereotypicality of the role name, with the morphological form of the word (the masculine) causing the development of the masculine representations (Gygax and Gabriel, 2011). These studies have shown that grammatical rules that determine the use or meaning of the masculine form and agreement rules can be difficult, if not impossible to obstruct. Moreover, the masculine grammatical gender has been shown to override not only semantic information, but also generic interpretation, as it is interpreted as a specific form. For example, *die Musiker* (the German word for musicians) which can be understood as masculine or generic, was automatically interpreted as masculine in Gygax et al. (2008). Most empirical research shows that masculine forms primarily elicit male-specific interpretations, compared to gender-balanced language alternatives that include the image of women (Gabriel and Mellenberger, 2004; Stahlberg et al., 2007; Gygax et al., 2008, 2009). Thus, when readers have two options for interpreting the masculine form, they usually resort to a specific interpretation of the form as masculine, thus evoking the male bias. In cases where grammatical gender information is not present, readers usually rely on gender stereotypes (Gygax and Gabriel, 2011).

In the present study, we used an event-related potential (ERP) technique to investigate the neural correlates of language processing. The ERP technique is used in neuroscience to measure sensory, cognitive, motor, and emotional processes in the brain (Kappenman and Luck, 2016). Electrophysiological measurements allow us to qualitatively separate different language processing mechanisms while monitoring language comprehension in real time (Kutas et al., 2006). Such tasks typically involve observing violations or anomalies based on introduced stimuli. A violation (deviation) in the form of incongruency refers to language stimulus that is usually unexpected in relation to the previous content (Frenzel et al., 2011). Of particular interest in the ERP signal are components that represent systematic and recurrent fluctuations in voltage over time (Kappenman and Luck, 2016).

The two most studied ERP components related to language processing are N400 and P600 (Kappenman and Luck, 2016). Much of the literature confirms that sentences containing semantically inconsistent words (e.g., “I like my coffee with cream and dog”) elicit the negative ERP component, which peaks approximately 400 ms after stimulation (N400) and has a centroparietal distribution (e.g., Kutas and Hillyard, 1980). Kutas and Hillyard (1980) were the first to identify N400 as a response to semantically anomalous words. They also found that the N400 component was greater after strong semantic mismatches than after moderate mismatches. Other studies (Siyanova-Chanturia et al., 2012; Misersky, 2017) have shown that the N400 reflects the interruption of sentence processing by a semantically inappropriate word and the reprocessing or an extra look that occurs when people try to understand a meaningless sentence. Thus, the N400 component is regulated by several factors, e.g., violation of semantic information and general knowledge, difficulty accessing information from semantic memory, semantically relevant but less expected information, lexical-semantic processing, etc., (Siyanova-Chanturia et al., 2012; Misersky, 2017).

The P600 is a positive-going ERP component that occurs approximately 600 ms after stimulus onset. P600 is associated with general sentence processing integration problems and is often observed in language manipulations involving grammatical complexity, grammatical ambiguity, or anomalies (Osterhout and Holcomb, 1992; Kaan and Swaab, 2003; Carreiras et al., 2004). Osterhout and Holcomb (1992) were the first to find that words that were inconsistent with the predictable/desirable sentence structure elicit a P600 potential (e.g., “The woman persuaded to answer the door”), which is quite different from the potential observed for contextually inappropriate words (N400). Similarly, Osterhout et al. (1997) found that sentences with pronouns containing violations of gender stereotypes (e.g., “The doctor prepared herself for the operation”) elicited greater positive potential (P600) than sentences with stereotypical matching pronouns (e.g., “The doctor prepared himself for the operation”). In general, the P600 has been used to identify syntactic problems (problems of agreement in time, gender, number, etc.) and responses to them–reanalysis or reintegration (Kaan and Swaab, 2003), “garden path” sentences, etc., (Siyanova-Chanturia et al., 2012).

We have based our research and interpretations on the presented theories of language information processing and comprehension. However, it should be noted that researchers’ opinions on how the N400 and P600 components are related to language processing may be conflicting, as their functional interpretation is still a matter of debate and their effects may also overlap (Delogu et al., 2019). In context of cognitive processing, recent theories have been discussing the N400 in connection to three different effects, namely access/retrieval, integration, and “hybrid” process (Kutas and Federmeier, 2000, 2011; Hagoort et al., 2004; Brouwer et al., 2012; Lau et al., 2016). While the N400 component has most often been attributed to the process of integration, recent studies (Kim and Osterhout, 2005; Kuperberg, 2007; Bornkessel-Schlesewsky and Schlesewsky, 2008; Kos et al., 2010; Brouwer et al., 2012, 2017) have shown that the N400 is more associated with retrieval processes in language comprehension. Similarly, these results show that P600 is not only associated with syntactic processes but has also been found to be related to semantic and pragmatic factors, in response to conflict manipulation/resolution, and to general integration difficulties (Kim and Osterhout, 2005; Kuperberg, 2007; Bornkessel-Schlesewsky and Schlesewsky, 2008; Kos et al., 2010; Brouwer et al., 2012, 2017). Uncertainty about the role of the N400 and P600 components is closely related to their often overlapping predictions (ambiguity about which factors cause which effects). This calls for studies with experimental designs that separate the retrieval and integration account of the N400 (e.g., Brown and Hagoort, 1993; Lau et al., 2009) and separate the syntactic from a non-syntactic account of the P600 (e.g., van Herten et al., 2005; Delogu et al., 2019).

Studies of language processing have been conducted in major European languages such as English, French, Italian, and German, however, this area of research has not yet been represented in neurolinguistic studies in the Slovene language. In the present study, we focused on grammatical aspects of the language and analyzed violations that occur after masculine grammatical gender and gender-balanced forms (feminine and masculine forms in word pairs). The study of Slovene language provides an important insight into the characteristics of processing gender information in a specific Slavic language, and at the same time represents an important contribution to existing ERP analyses on languages with grammatical gender. The morphological complexity of Slovenian provides the opportunity for many research areas and methods and allows comparison with Slavic languages, other languages with grammatical gender, and languages with natural gender markers (e.g., English).

In Slovene language, as in most other languages with grammatical gender, the masculine grammatical gender plays the role of a neutral (gender unmarked) or generic gender. Such dual use of the masculine grammatical gender refers to the masculine gender and, at the same time, to persons whose gender is unknown or insignificant, or to a group of people consisting of both genders. This leads to a semantic ambiguity that is usually resolved in favor of men (Gabriel et al., 2008). To address this issue, in the present study we examined how (masculine) grammatical gender affects our understanding of differently gendered referents (Misersky, 2017), in comparison to gender-balanced forms, consisting of feminine and masculine forms in a word pair. In half of all experimental material, the masculine generic role name (e.g., surgeons _masculine form_) was followed by a feminine/masculine verb (e.g., spend _feminine/masculine verb_), and in the other half, the gender-balanced role names (e.g., surgeons _feminine form_ and surgeons _masculine form_) were followed by a feminine/masculine verb (e.g., spend _feminine/masculine verb_). Many studies have shown that masculine grammatical gender in role names leads to male specific interpretations, raising the question of whether masculine gender is appropriate as a “gender neutral” form. This has been increasingly investigated in a number of experimental studies since the early 1970s in English language and later in the 1990s for other languages (e.g., Stahlberg and Sczesny, 2001; Braun et al., 2005, etc.). However, not many ERP studies have addressed the inclusivity of gender-balanced forms, which was the focus of this study.

Based on theoretical findings about the use and effects of the masculine generic form, we expected that the role noun in the masculine generic form would elicit predominantly masculine interpretations. In contrast, we expected the use of a gender-balanced form to produce different effects, open to masculine and feminine interpretations.

Based on these expectations, we designed the following hypotheses:

(1) In cases where the antecedent in the masculine generic form is followed by a verb in the feminine form, a violation of expectations will be triggered in the processing of the sentence. We expected that if the violation is perceived as a syntactic violation, P600 will be evoked. When the violation is perceived as a semantic violation, N400 will be evoked.
(2) In cases where the antecedent in the masculine generic form is followed by a verb in the masculine form, no violation of expectations in the processing of the sentence will be triggered. We did not expect ERP components related to semantic (N400) or syntactic violations (P600) to occur in this condition.
(3) In cases where the antecedent in gender-balanced form is followed by a verb in feminine or masculine form, no violation of expectations in the processing of the sentence will be triggered. We did not expect ERP components related to semantic (N400) or syntactic violations (P600) to occur in this condition.

## Materials and methods

### Preregistration

The study was preregistered on Open Science Framework in November 2019.^1^ Unless otherwise stated, all procedures were planned during preregistration.

Electroencephalographic (EEG) recordings were performed from November 6, 2019, to January 16, 2020, at the Department of Neurology (University Medical Center) in Ljubljana, Slovenia.

### Language

The study was conducted in Slovene language, a Slavic language, which is a morphologically complex language that uses grammatical gender as a morphological category (Toporišič, 1984). Although in Slovenian every noun is marked with gender (gender is also marked on adjectives, verbs, articles, suffixes, and various types of pronouns) and it is necessary to learn gender along with the word (Toporišič, 1976), in some words gender can also be biologically motivated, which means that it is associated with the “natural” or referential gender and thus expresses the semantic component “feminine”/“masculine.” Such a match between the “natural” and grammatical gender is characteristic for personal names (proper names, e.g., Petra, Jože; kinship, e.g., father, mother) and role names (zdravnik–male doctor; zdravnica–female doctor) (Toporišič, 1992; Bešter, 1997; Marušič, 2018).

In compound subjects (feminine and masculine pairs of words as a gender-balanced form), the order of the gender of the subject is especially important. Agreement rules in Slovenian show that the verb prefers to match a noun that is closer to it (Marušič, 2005); however, practice also highlights the importance of hierarchy in agreement (Willer-Gold et al., 2016). The study by Willer-Gold et al. (2016), which analyzed gender agreement (in nouns and verbs) in South Slavic languages (including Slovenian), showed that the grammatical genders (masculine, feminine, and neuter) have different potentials to control/determine agreement, with the masculine having the greatest power to determine agreement (Willer-Gold et al., 2016). In cases where the noun was presented first in the feminine gender and then in the masculine gender, 97% of participants rated the masculine form of the verb as appropriate and only 3% did so in the case of the feminine form. When the gender order was reversed and the noun was presented first in the masculine form and then in the feminine form, 75% of the participants still chose the masculine form of the verb as correct and only 25% chose the feminine form (Willer-Gold et al., 2016). The study by Willer-Gold et al. (2016) focused on noun-verb correlation using nouns describing inanimate objects rather than people (e.g., bell, books; here, grammatical gender has nothing to do with natural gender, i.e., it is merely a grammatical category). Our study has the potential to contribute to these findings by using personal nouns (role names) in gender-balanced forms, which are somewhat more complex to understand because they are influenced by both linguistic (syntax) and non-linguistic reality (role names as female/male).

Due to the specific characteristics of the Slovenian language, we had to deviate from the model of previous studies, which was based on English, German, French and Italian, in order to obtain a sense of naturalness in reading. The main deviation from previous studies was the placement (and measurement) of gender in the verb instead of the noun. In the previously mentioned studies, nominal anaphora (i.e., she, him) was used to refer to the noun. The use of the anaphoric verb is a novelty, but also something that could potentially have different implications than noun or pronoun marking. There was a possibility that the gender marking of the verb we used as anaphora was considered less obvious (compared to nouns/pronouns), which could lead to a problem in determining the direct connection between the anaphoric verb in the second sentence and the antecedent presented in the first sentence (since other verbs also occur in between).

### Participants

Twenty-six participants (17 females, 9 males) with a mean age of 20.1 years (range: 18–23) and a mean length of education of 13.6 years (range: 12–17) participated in the study. Twenty-four participants were right-handed. All participants were native Slovenian speakers and had a normal or corrected-to-normal vision.

Five participants were excluded from all EEG analyzes: four due to an excessive number of excluded channels and one due to an excessive number of excluded epochs. We recorded one more participant than planned during preregistration.

The study was approved by the Ethics Committee of the Faculty of Social Sciences, University of Ljubljana in January 2019. All participants signed an informed consent form before participating in the study and received reimbursement for their participation in the form of a coupon for 10 euros. The exact aim of the study was not revealed to the participants until the end of the experiment.

### Design and materials

Participants were presented with a general statement about an occupational group consisting of two sentences in Slovenian. We used a match-mismatch paradigm in which participants had to judge whether a second sentence was a meaningful continuation of the previous sentence.

The coordinate clauses were inspired by previous research (Gygax et al., 2008; Irmen et al., 2010). In all sentences a group of people–an occupational role–was introduced in the subject position, e.g., *kirurgi* (surgeons); *cvetličarji* (florists). In preparing the material, we considered both a set of stereotypical male and female occupations in order to balance the sample; however, the stereotypicality of the role names was not measured. These role names served as the antecedent of a gender specifying verb that had no formal disagreement with the antecedent. The second sentence began with a quantifier that emphasized that the group was not exclusively made up of men or women, e.g., nekaterim/mnogim (to/for some/many/most), and then introduced a verb in either feminine or masculine form (e.g., so prišle/prišli; in English: they came–feminine form or they came–masculine form). The measured verb occurred in various places in the second sentence, but in most cases it was presented at the end of a second sentence. The verb was marked with either the feminine or masculine grammatical gender. Half of the antecedents were in the masculine plural form, the most conventional and general way of writing (gender-mixed) role names in Slovenian; half of the antecedents were in the gender-balanced form (using both the feminine and masculine grammatical forms). For the gender-balanced forms, we chose an order of nouns that is more common in Slovenian (in terms of agreement rules) so that it would sound as natural as possible to the participants.

Examples of sentences for each condition (measured verbs/anaphors are written in italic):

(1) Generic masculine form + feminine verb:
Kirurgi_(masculine form)_ so ponovno delali nadure. Večini je bilo to odveč, saj bi svoj čas raje *preživljale*_(feminine verb)_ s partnerjem.
English: Surgeons_(masculine form)_ worked overtime again. For most, this was unnecessary as they would rather *spend*_(feminine verb)_ their time with their partner.

(2) Generic masculine form + masculine verb:
Telefonisti_(masculine form)_ so si med pavzo privoščili kavo. Ker je bila nekaterim pregrenka so jo *pili*_(masculine verb)_ z mlekom.
English: The telephonists_(masculine form)_ had coffee during the break. Because it was,
too bitter for some, they *drank*_(masculine verb)_ it with milk.

(3) Gender-balanced form + feminine verb:
Skladateljice_(feminine form)_ in skladatelji_(masculine_ _form)_ so se navadili samostojnosti pri svojem delu. Mnogim se je zato zgodilo, da so se skupinskega dela popolnoma *odvadile*_(feminine verb)_.
English: Composers_(feminine form)_ and composers_(masculine_ _form)_ are accustomed to autonomy in their work. It happened to many that they were completely *disengaged*_(feminine verb)_ from group work.

(4) Gender-balanced form + masculine verb:
C̀istilke_(feminine form)_ in Čistilci_(masculine_ _form)_ so imeli pozno malico. Mnogim je to ustrezalo, saj so si lahko *vzeli*_(masculine verb)_ več časa zanjo.
English: Cleaners_(feminine form)_ and cleaners_(masculine form)_ had late lunch. That suited many, because they could *spend*_(masculine verb)_ more time on it.

In order for sentences to appear more conventional and genuine, we formed them in the past tense.

Participants were presented with a total of 310 sentence pairs, 50 sentence pairs for each of the four experimental conditions. In addition, 110 filler sentence pairs (half in the masculine generic form, half in the gender-balanced form) were included that contained either a semantic anomaly (50 sentences; in the generic and gender-balanced form) or a syntactic anomaly (50 sentences; in the generic and gender-balanced form) in the position of the anaphoric verb, or were presented without reference to gender in the verb, i.e., in the plural form (10 sentences; in the generic and gender-balanced form). Filler sentences were used to reduce predictability in reading. Sentences with semantic and syntactic anomalies were also used as control conditions, as they were expected to trigger N400 or P600. Sentences were presented in randomized order.

Examples of filler sentences:

(1) Semantic anomaly:
Natakarji so se sprli med sabo. Mnogim je prekipelo, zato so na glas *šepetali*.
English: Waiters have been arguing. Many were overwhelmed, so they loudly *whispered*_(*semanticallyincorrect*_ _*verbform*)_.

(2) Syntactic anomaly:
Mizarji so sprejeli preveliko naročilo. Mnogim je bilo zato naporno, ker so *hitiš* celo noč z delom.
English: The carpenters accepted a big order. This was exhausting to many, because they *were in a hurry*_(syntactically incorrect verb form)_ working all night.

(3) Plural form:
Pilotke in piloti imajo med leti včasih premalo časa za počitek. Večini se zato ne zdi smiselno, da v vmesnem času *zaspijo*.
English: Pilots_(feminine form)_ and pilots_(masculine form)_, have sometimes too little time to rest between flights. Most, therefore, do not find it sensible *to fall asleep*_(indefinite plural form)_ in the meantime.

The participants’ task was to indicate with “yes” or “no” whether the sentence containing the anaphora in the verb is a logical continuation of the sentence containing the antecedent after each statement.

The experimental material was designed in such a way that all sentences should be assessed as logically related (except in the case of intentional syntactic and semantic anomalies). However, the complexity of agreement rules in Slovenian (as shown by Willer-Gold et al., 2016) could also lead to feminine verbs being understood as violations. We addressed this assumption in two ways: (1) by presenting all role nouns in the plural form (generic and word pairs), thereby attempting to indicate that the masculine generic or gender-balanced form refers to a group of (gender-mixed) people, which allows for gender-inclusive interpretations; (2) by designing (second) sentences with the use of quantifiers that additionally confirm that we are referring to a group of people. By using quantifiers (e.g., “for some of them”), we aimed to increase the inclusivity of feminine verbs by referring specifically to some of the people in the group (assuming that participants understood this role noun to refer to a group of women and men).

When we form sentences in which role nouns represent a gender-mixed group of people and explicitly mention that we are referring to “many/some of them” the use of the feminine verb should not be considered inappropriate. Thus, the grammatical incongruity could be understood (1) as a biased effect of the masculine gender as a generic, causing predominantly male associations, or (2) as a result of violating rules of agreement that could influence the masculine form having a greater potential to match the verb in the masculine form (even if understood as generic). By creating an obvious image of men and women in the gender-balanced forms, we expected that they might cause greater acceptance of the agreement of a verb in the feminine form.

### Procedure

Participants were tested individually in a small, quiet room. Sentences were presented in blocks of one to a maximum of three words, depending on the length of the word. The names of the occupational roles (antecedents) and the verbs expressing gender were always presented independently. Before the beginning of the sentence, a blank screen was displayed for 1,500 ms, followed by a word display for 600 ms, then a blank screen of 500 ms until the next word. At the end of the sentence, the blank screen followed for 1,000 ms, and then a question mark appeared for participants to decide whether the second sentence was a meaningful continuation of the first. For each pair of sentences, participants had to press a button to answer “yes” or “no.” Participants were asked to make a quick decision based on their first impression. After the response, the question mark color changed to blue for 500 ms, followed by the inter-trial interval. To reduce expectation effects and line noise in the averaged ERPs, the length of the inter-trial interval varied randomly from 1,400 to 1,600 ms.

Sentences were displayed in Helvetica font in black color on a gray background at eye level, approximately 70 cm in front of the participant. The stimuli were presented on a 24” LCD screen with a refresh rate of 120 Hz. Stimuli spanned 2.6° of vertical visual angle and up to 29° of horizontal visual angle. The task was prepared in PsychoPy 3 (Peirce et al., 2022). A Cedrus keyboard (Cedrus Corporation, San Pedro, CA, USA), model RB-540, was used to record the behavioral responses.

The procedure lasted between one and a half and 2 h per participant. It was divided into seven sections (each lasting approximately 10–15 min) and six breaks for participants in between.

### Electroencephalographic recording

EEG activity was recorded with the BrainAmp MRplus amplifier (resolution: 0.5 μV, sampling rate: 500 Hz, bandpass filter: 0.1–250 Hz with slope 12 dB/octave; Brain Products, GmbH, Germany) connected to a 64-channel EEG cap with electrodes arranged according to the extended 10-10 system. We used two types of caps: ActiCap (Brain Products) with active Ag/AgCl electrodes and BrainCap TMS (BrainProducts) with passive Ag/AgCl electrodes. The use of BrainCap was not planned in the preregistration, but we decided to use it, because it allowed us to use different cap sizes and thus ensure a better signal. The EEG signal was recorded using a PC running BrainVision Recorder 2.04 (Brain Products, GmbH, Germany). FCz was used as reference and AFz as ground electrode.

### Data analysis

#### Behavioral data analysis

Given that the study was primarily designed to examine ERP effects, the analysis of behavioral data was not planned at preregistration.

Responses (“yes” vs. “no”) were compared using a generalized linear mixed effects model for binary data (i.e., binomial logistic regression).

We made five comparisons: (a) masculine vs. feminine verb, (b) feminine verb vs. semantic error, (c) masculine verb vs. semantic error, (d) feminine verb vs. syntactic error, (e) masculine verb vs. syntactic error. All models included grammatical form (gender vs. gender-balanced) as a fixed effects factor and participant as a random effects factor. Because these comparisons were not planned at preregistration, we used the Benjamini-Hochberg correction to control for false discovery rate (FDR-BH) for all tests combined (Benjamini and Hochberg, 1995). Statistical significance was assessed at the significance level *q* = 0.05. Analysis was performed using the R package lme4 (Bates et al., 2015).

#### Electroencephalographic preprocessing

EEG preprocessing was performed using Matlab 2018a (Mathworks, Inc.), EEGLAB (Delorme and Makeig, 2004), ERPLAB (Lopez-Calderon and Luck, 2014), and customized scripts. To enable preprocessing, virtual electrooculogram (EOG) channels were calculated as the difference between channels F7 and F8 for horizontal movements and as the average of channels Fp1 and Fp2 for vertical movements. The preprocessing pipeline included: (1) high-pass filtering of the non-epoched data at 0.1 Hz (2nd order Butterworth, slope: 12 dB/octave), (2) CleanLine algorithm to attenuate 50 Hz line noise (Mullen, 2012) (3) epoching between −200 and 1,100 ms, time-locked to stimulus presentation, (4) visual inspection of epochs and rejection of epochs with gross artifacts (e.g., muscle noise), (5) visual inspection of channel and rejection of bad channels, (5) automatic rejection of bad epochs if they exceed a *z*-score of 2.3 for root mean square difference, variance, or amplitude compared to all other epochs, (6) ICA decomposition using the AMICA algorithm (Palmer et al., 2012). Independent components (ICs) were manually inspected and rejected based on the following criteria: high correlation with EOG channels and frontal scalp distribution (indicating blinks or eye movements), low signal-to-noise ratio, high power at 50 Hz frequency activity located on individual channels (line noise), high power high frequency activity (muscle artifacts). ICA was followed by interpolation of the missing channels using spherical spline interpolation. ERPs were baseline corrected for the period before stimulus presentation. In a final step, the data were filtered with a low-pass filter, with the lower edge of the frequency pass band at 60 Hz (2nd order Butterworth, slope: 12 dB/octave) for visualization purposes only.

#### Electroencephalographic data analysis

We performed two types of statistical analyzes: (1) an analysis based on mean amplitudes, and (2) mass univariate statistical analyzes. The analyzes based on mean amplitudes allowed us to test hypotheses related to the P600 and N400 potentials using analysis of variance, which allowed us to test for interactions between factors. On the other hand, mass univariate analyzes allowed us to explore effects outside the time windows and channels in which the potentials of interest were expected to occur.

For the analysis based on mean amplitudes, we first defined time windows. In the preregistration, we planned to determine time windows as ± 100 ms from the peak of the component on global field potential. However, due to the overlap of different components, this procedure turned out not to be feasible, so we selected time windows based on the expected latencies of the components. Thus, the time window was 300–500 ms post stimulus for N400 based on the semantic error condition and 500–700 ms post stimulus for P600 based on the syntactic error condition. These latencies correspond to the observed peaks of the components.

Analysis was performed on nine electrodes in frontal, central and posterior positions at the midline, left and right hemispheres (F3/z/4, C3/z/4, P3/z/4). Two ANOVAs were performed, one for the N400 and one for the P600 potential. The factors for the repeated measures ANOVA were (1) gender of the antecedent (female, male), (2) grammatical form (generic, gender-balanced), (3) laterality (left, medial, right), (4) anteriority (anterior, central, posterior). In case of violation of the sphericity assumption, Greenhouse-Geisser correction was used. Statistical significance was assessed at the significance level α = 0.05. For brevity, only information on the effects of gender and grammatical form and their interaction are mentioned in the main text; further details are provided in Table 1. Analysis was performed using the R package ez (Lawrence, 2016), plots were created using the R package ggplot2 (Wickham, 2016).

**TABLE 1:** Analysis of variance of mean amplitudes for N400 (300–500 ms) and P600 (500–700 ms) with the factors gender (male, female), grammatical form (generic, gender-balanced), anteriority (frontal, central, parietal), and laterality (left, midline, right).

	N400 (300–500 ms)					P600 (500–700 ms)				
Factor	*df1*	*df2*	*F*	*p*	< 0.05	*df1*	*df2*	*F*	*p*	< 0.05
intercept	1	19	6.02	0.024	*	1	19	21.90	<0.001	*
gender	1	19	0.09	0.774		1	19	21.55	<0.001	*
grammatical form	1	19	0.33	0.571		1	19	0.90	0.356	
anteriority	2	38	30.97	<0.001	*	2	38	41.13	<0.001	*
laterality	2	38	2.66	0.105		2	38	2.62	0.086	
gender × grammatical form	1	19	0.09	0.771		1	19	0.42	0.526	
gender × anteriority	2	38	2.97	0.082		2	38	29.66	<0.001	*
grammatical form × anteriority	2	38	4.52	0.033	*	2	38	7.01	0.010	*
gender × laterality	2	38	1.13	0.335		2	38	7.42	0.002	*
grammatical form × laterality	2	38	0.53	0.595		2	38	0.13	0.881	
anteriority × laterality	4	76	4.89	0.001	*	4	76	2.47	0.052	
gender × grammatical form × anteriority	2	38	3.29	0.048	*	2	38	1.48	0.241	
gender × grammatical form × laterality	2	38	0.83	0.442		2	38	1.17	0.322	
gender × anteriority × laterality	4	76	0.57	0.607		4	76	1.33	0.266	
grammatical form × anteriority × laterality	4	76	0.52	0.628		4	76	0.66	0.554	
gender × grammatical form × anteriority × laterality	4	76	1.89	0.138		4	76	0.98	0.406	

*p < 0.05.

Mass univariate analyses were performed using the Mass Univariate ERP Toolbox (Groppe et al., 2011) for the time range from 200 to 1,000 ms on all channels. BH-FDR correction (Benjamini and Hochberg, 1995) was used and statistical significance was assessed at significance level *q* = 0.05. The following planned pairwise comparisons (*t*-tests) were performed: (1) generic grammatical form, feminine verb vs. generic grammatical form, masculine verb, (2) generic grammatical form, feminine verb vs. gender-balanced grammatical form, feminine verb, (3) generic grammatical form, feminine verb vs. gender-balanced grammatical form, masculine verb, (4) generic grammatical form, feminine verb vs. syntactic violations (control condition), (5) generic grammatical form, feminine verb vs. semantic violations (control condition). In addition, we performed the following comparisons: (6) gender-balanced, feminine verb vs. semantic violations, and (7) gender-balanced, feminine verb vs. syntactic violations. These additional comparisons were not planned, because N400 or P600 potentials were not expected to occur in the gender-balanced, feminine verb condition. We decided to perform these comparisons after observing a prominent P600 in this condition.

Clusters of significant differences spanning less than 10 ms or less than 3 channels were not interpreted as significant, because the BH-FDR correction only guarantees that no more than proportion *q* of rejected null hypotheses are false and such small effects are more likely to be falsely rejected null hypotheses.

## Results

### Behavioral results

**FIGURE 1:**
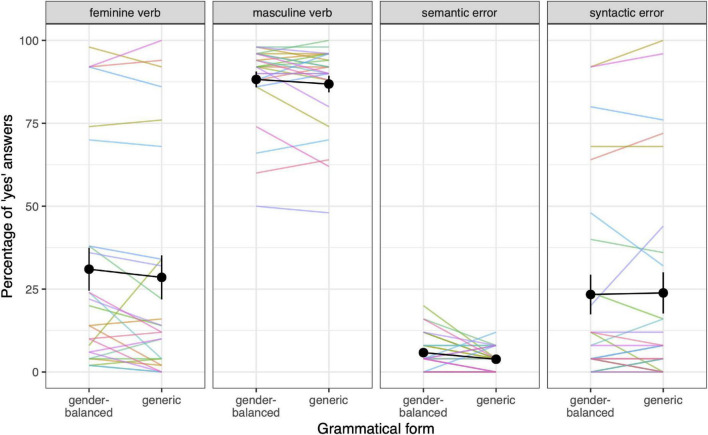
Behavioral responses–answers to the question of whether the second sentence is a meaningful continuation of the first sentence. Each color represents one participant, the black color represents the mean across participants, and the error bars represent the standard error. On average, participants rated feminine verbs, syntactic errors, and semantic errors as non-meaningful continuations of the first sentence, while masculine verbs were rated on average as a meaningful continuation. There were no differences in terms of grammatical form.

In the behavioral analysis, we used logistic regression to test whether responses were significantly different between the different types of anaphoric verbs and both grammatical forms (see Supplementary Table 1 for detailed results). Responses were significantly different between the types of anaphoric verbs in all five comparisons (masculine vs. feminine verb, feminine verb vs. semantic error, masculine verb vs. semantic error, feminine verb vs. syntactic error, masculine verb vs. syntactic error), but the magnitude of the differences varied. As can be seen in Figure 1, in the masculine verb condition, participants rated the second sentences as meaningful continuations of the first sentence in about 85% of cases on average, followed by the feminine verb and syntactic error with about 30% of the responses “yes.” Participants rated semantic error cases almost exclusively as non-sensible continuations. There were no statistically significant differences in responses between gender-balanced and generic forms in any of the comparisons. The interaction between anaphoric verb type and grammatical form was not significant in any of the models. On the other hand, the variability of responses between participants across types of anaphoric verbs varied greatly. Responses varied most in the feminine verb and syntactic error conditions; however, patterns of responses within participants were very similar in these two conditions. There was also some variation in responses to verbs in masculine gender, but very little variation in responses to semantic violations.

### Event-related brain potentials

**FIGURE 2:**
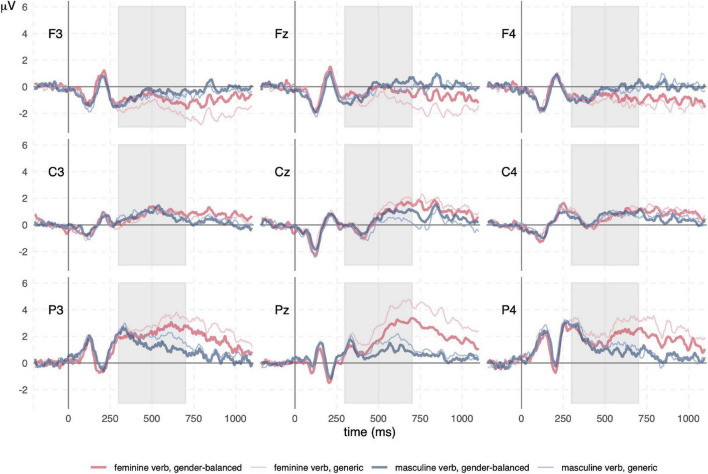
Event-related potential (ERP) waveforms for comparison of feminine and masculine verbs in generic and gender-balanced condition. The early potentials (up to 250 ms) are similar in all conditions and are followed by N400 in the time window from 300 to 500 ms. N400 is followed by P600, which is visible only in both conditions for the feminine verb and is larger in the feminine verb, gender-balanced condition. The differences in N400 between the feminine verb, gender-balanced and feminine verb, generic conditions can be attributed to the differences in P600 (see also Figure 4). The gray rectangles represent the time windows used to calculate the mean amplitude.

**FIGURE 3:**
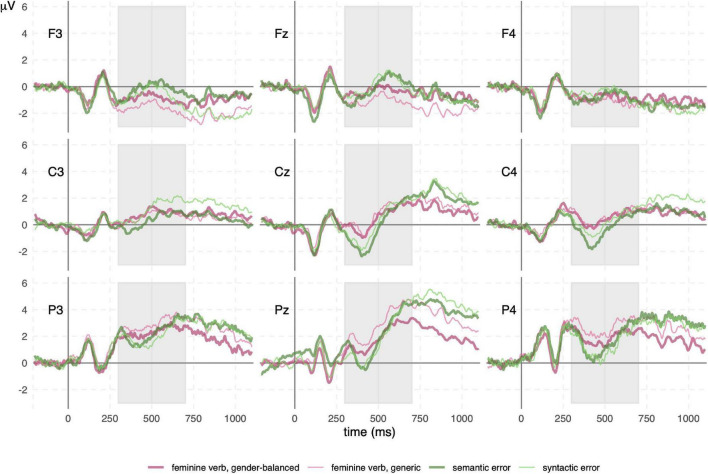
Event-related potential (ERP) waveforms for comparison of feminine verbs and control conditions. N400 and P600 are present in all conditions, although to varying degrees. Semantic and syntactic error conditions have very similar waveforms. The N400 is larger in both control conditions compared to both feminine verb conditions. The P600 is similar in both control conditions and in the feminine verb, gender-balanced condition, but smaller in the feminine verb, generic condition. The gray rectangles represent the time windows used to calculate the mean amplitude.

We observed early visual evoked potentials in all conditions: P1 at 110 ms in the parietooccipital area was followed by N1 in parietal channels and P2 in frontocentral channels, both around 200 ms (Figures 2, 3). Regardless of the presence of N400 or P600, another potential with positive amplitude was observed in parietooccipital channels at approximately 870 ms. Since this potential was present in all conditions, it was most probably related to the disappearance of the visual stimuli from the screen.

**FIGURE 4:**
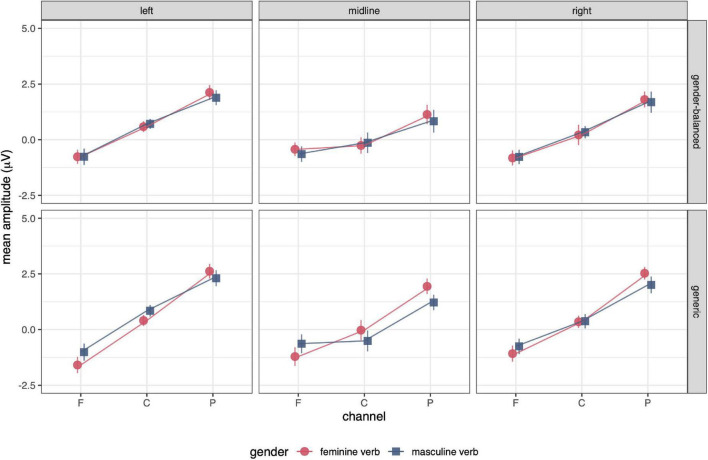
Mean amplitudes of the N400 in the time window 300–500 ms post stimulus for feminine and masculine verb, gender-balanced, and generic conditions. There was no effect of gender or grammatical form on the mean amplitudes in this time window. The error bars represent the standard error.

**FIGURE 5:**
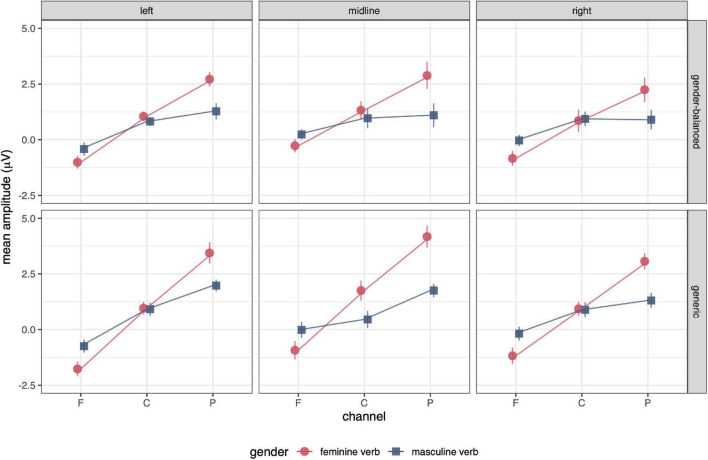
Mean amplitudes for P600 in the time window 500–700 ms post stimulus. The amplitude gradient from anterior to posterior channels is very small in both masculine verb conditions, indicating the absence of P600 in these conditions (see also Figure 2). ANOVA showed a significant effect of gender and grammatical form × anteriority interaction, indicating a smaller effect of P600 in the gender-balanced condition. The error bars represent the standard error.

**FIGURE 6:**
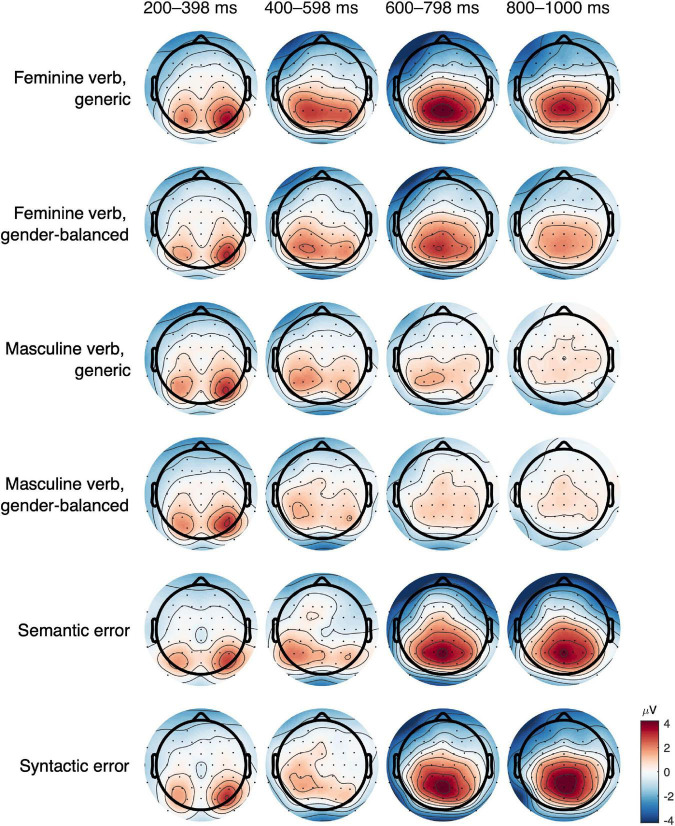
Topographic plots of all conditions grouped in time ranges of 200 ms from 200 to 1,000 ms post stimulus.

**FIGURE 7:**
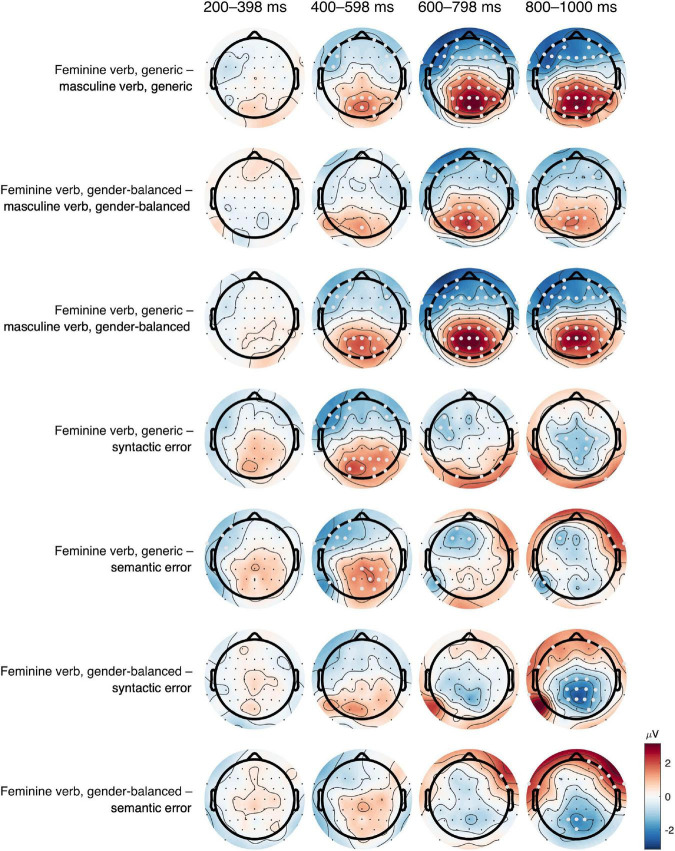
Results of mass univariate tests–topographic plots for all comparisons. Results were averaged in time windows of 200 ms for easier visualization; see Supplement for raw results of mass univariate tests. White dots represent statistically significant differences (FDR-corrected, *q* < 0.05). There were large differences between feminine verb, generic and both masculine verb conditions, starting at 200 ms, mainly centroparietal, corresponding to the difference in P600. Equal, but smaller differences are observed between the feminine verb, gender-balanced, and the masculine verb, gender-balanced condition. The differences between the feminine verb, generic, and both control conditions (syntactic error, semantic error) are present in the time window 400–598 ms, corresponding to a larger N400 in both control conditions. The comparison between feminine verb, gender-balanced, and both control conditions revealed a larger amplitude in both control conditions, corresponding to a larger P600 in late time windows in both control conditions.

ERP results are presented in the following order: ERP waveforms are shown in Figures 2, 3; mean amplitudes related to N400 and P600 are shown in Figures 4, 5, respectively; topographies and results of mass univariate tests are shown in Figures 6, 7.

#### N400

The N400 potential had the highest (negative) amplitude in the midline and right central channels (Figures 2, 3, 6) from 300 to 500 ms. The N400 potential was most prominent in both control conditions (semantic and syntactic error), but it was also observed in all other conditions. Mass univariate tests revealed significant differences between both feminine verb conditions vs. semantic error (Figure 7; Supplementary Figures 4, 8) and between the feminine verb condition vs. syntactic error (Supplementary Figures 5, 6) in the time window from 300 to 500 ms.

This difference in the time window from 300 to 500 ms was largest when comparing feminine verb, generic vs. syntactic error, followed by feminine verb, generic vs. semantic error. The difference between feminine verb, gender-balanced condition, and semantic error was rather small in this time window, and there were no differences between feminine verb, gender-balanced condition, and syntactic error. For all significant differences, the difference was negative for left frontal channels and positive for right parietooccipital channels (Figure 7).

Analysis of variance of mean amplitudes for the 300–500 ms time window revealed no main effect of gender on N400 amplitude (Table 1; Figure 4). There was no significant effect of grammatical form and no interaction between gender and grammatical form. There was a significant effect of grammatical form × anteriority and gender × grammatical form × anteriority interaction. The amplitude was less bipolar (higher in frontal channels and lower in parietal channels) for the gender-balanced condition, indicating a smaller N400 effect in this condition, but the effect of the interaction was not large.

#### P600

P600 had the highest amplitude in the midline parietal channels with effects spreading throughout the parietal lobe bilaterally from 500 to 1,000 ms (Figures 2, 3, 6). P600 was present in both feminine verb conditions and both control conditions, but it was absent in both masculine verb conditions.

Mass univariate tests revealed small differences between both feminine verb conditions and both control conditions in the time window from 600 to 1,000 ms (Figure 7, Supplementary Figures 4–7). The differences were largest when comparing the feminine verb, gender-balanced, and both control conditions. The P600 amplitude in the centroparietal channels was smaller in the feminine verb, gender-balanced condition compared to both control conditions.

Comparisons of mean amplitudes for feminine vs. masculine verb conditions from 500 to 700 ms revealed large significant differences that were most pronounced at the centroparietal electrodes (Figure 7, Supplementary Figures 1–3). Both ANOVA (Table 1) and mass univariate tests confirmed that P600 was present in both feminine verb conditions and absent in both masculine verb conditions, with feminine verb, generic condition vs. all other conditions showing the largest effects and feminine verb, gender-balanced vs. masculine verb, gender-balanced showing the smallest effects (Figures 5, 7). There was also a significant interaction between grammatical form and anteriority, indicating a smaller effect of P600 in the gender-balanced condition.

## Discussion

In the present study, we investigated how masculine grammatical gender and gender-balanced forms as role nouns affect the processing of referents in Slovenian. Our hypotheses defining various effects of the link between the grammatical form (or forms) of the role name and the gender of the verb were only partially confirmed. One of the most important findings of our study is that the masculine generic form was interpreted differently from the gender-balanced forms. Following both the gender-balanced and masculine generic forms, P600 (but not N400) was observed in response to the feminine verb, but not to the masculine verb. However, the P600 amplitude was smaller for gender-balanced form than for the masculine generic form. Some possible interpretations for the obtained results are presented below.

### Feminine verb after masculine generic form is perceived as syntactic violation

In the first hypothesis we assumed that when the violation is perceived as a syntactic violation, the effect of P600 is observed, and when the violation is perceived as a semantic violation, the effect of N400 is observed. P600, but not N400, was observed in response to the feminine verb. This can be interpreted to mean that the feminine verb was perceived as a syntactic (rather than semantic) violation after the masculine (generic) form.

The electrophysiological response to the feminine verb indicates that participants had difficulty when attempting to integrate female referents with the masculine grammatical form, suggesting that participants perceived the sentence as meaningless and/or syntactically incorrect (Osterhout and Holcomb, 1992). There are several possible reasons for this. Since we only found P600, we can assume that the violation is a result of the problems in agreement of masculine (noun) and feminine (verb) genders, since the masculine noun has a preference for matching the masculine verb. This is despite the fact that the grammatical rules clearly state that the feminine continuation after the masculine generic form is correct when it is clear that we are referring to a mixed-gender group of people. We have tried to achieve this with various strategies in (second) sentence formation. In the context of this interpretation, there is also the possibility that participants, having encountered a verb in feminine form (we used the feminine verb to refer to women as part of the group), expected the role noun to be in feminine form, again because of gender agreement rules and the narrow/specific interpretation of the feminine verb (referring only to women).

It is also significant that we found no N400 effect, which would indicate an effect of a purely semantic nature. We believe that the reason we obtained the P600 but not the N400 effect is closely related to (1) the way the experiment was conducted and (2) the measurement of anaphora in the verb (instead of noun/pronoun). By not giving participants much information about the study and asking them to judge whether the second sentence was a meaningful continuation of the first sentence (which could be understood as a task in which they had to judge the grammatical correctness of the sentence), and also by the way the task was conducted (participants read the sentences word by word), participants could be motivated to focus more on the words and syntactic correctness and ignore the contextual (semantic) dimension. Both aspects of the experiment are consistent with other similar experiments. The other possible reason for the absence of the N400 is the use of the anaphoric verb. It is possible that activation of the N400 requires explicit gender emphasis (achieved by the noun/pronoun) and that the anaphoric verb was too subtle in this regard, leading to the perception of an insufficient connection between the antecedent and the anaphora.

We should also keep in mind that newer literature (e.g., Delogu et al., 2019) suggests that the role of N400 and P600 is uncertain and that different language features overlap (i.e., an interaction of semantic and syntactic processing), which leads to considering P600 and N400 as a continuum and not easily separable. Thus, P600 has been associated not only with syntactic processes but also with semantic and pragmatic factors, indicating general integration difficulties (e.g., Kos et al., 2010; Brouwer et al., 2012, 2017). In addition to understanding our results as a purely syntactic anomaly, these theories offer the possibility of understanding feminine verbs (simultaneously) as semantic violation and interpreting the masculine form as referring only to a group of males. The latter interpretation would be consistent with the results of our behavioral analysis, which found no major differences between syntactic and semantic anomalies. However, based on our study, it would be difficult to draw such conclusions, so we present this only as a conjecture. Nonetheless, this represents a research direction that should definitely be pursued in the future.

### Masculine verb after masculine generic form is not perceived as violation

In the second hypothesis, we predicted that in cases where the antecedent in the masculine generic form is followed by a verb in the masculine form, no violation of expectations would be triggered in the processing of the sentence. We expected that ERP components related to semantic (N400) or syntactic violations (P600) would not occur in this situation. This prediction was confirmed, as the N400 or P600 effects were not observed. These results were consistent with the results of the behavioral analysis, in which the verb in the masculine form was perceived as congruent with the role noun in most cases. The masculine gender of the verb after the antecedent in masculine form met participants’ expectations (the agreement of semantic and grammatical gender and/or the congruence with agreement rules) and reaffirmed the narrow functioning of the masculine generic form (as exclusively masculine).

### Feminine verb following the gender-balanced form is perceived as a smaller syntactic violation compared to masculine generic form

In the third hypothesis, we predicted that mental representations of women will be higher for gender-balanced forms (word pairs) than for masculine generics. We therefore expected that event-related potentials (P600 or N400) would not be observed. This prediction can be partially confirmed. We observed P600 in response to feminine verbs in the gender-balanced condition, but the amplitude of P600 was smaller than in the generic condition. We did not observe P600 or N400 in response to masculine verbs in the gender-balanced conditions.

These results confirm the hypothesis that the gender-balanced form is more acceptable for feminine verbs than the generic form. However, even after the gender-balanced form, the feminine verb does not seem to be fully congruent with the antecedent, as it still triggers the P600 effect. There are several possible reasons for this result. Although we explicitly mentioned the role name in feminine form (as part of a word pair, along with the masculine form), the use of these forms is still a relatively new linguistic practice, so there is a possibility that many participants found these forms as unusual. The masculine forms are overwhelmingly used in practice and perceived as more correct, as they are recognized as appropriate even in cases where the masculine gender is not even part of a composite subject (see Willer-Gold et al., 2016). It could also be that (as mentioned in the discussion of the first hypothesis) when participants encountered feminine verbs, they expected feminine forms in a role name and were only partially surprised because the feminine form was present but alongside the masculine form. Based on the observed P600 effect, we can speculate that the violations of expectations were related to syntactic factors, such as violation of agreement rules, where the feminine noun in the gender-balanced form somehow influenced a smaller syntactic violation even though it was placed at the beginning of the sentence and had no direct effect on the closeness or hierarchy rule. This was confirmed by a behavioral analysis in which feminine verb forms were treated similarly to syntactic errors.

However, the behavioral analysis also showed that feminine forms of the verb were mostly (in about 70%) seen as incongruent with the antecedent, regardless of the grammatical form of the antecedent. The masculine noun was always closest to the anaphoric verb in both cases of role naming (masculine generic form and gender-balanced form), which led us to believe that we should expect the same type and intensity of violation response to the feminine verb, but this was not the case. Therefore, we also consider it possible that the explicit mention of feminine nouns in the gender-balanced form contributed to a higher mental expectation of women (compared to the masculine generic form), which might have raised not only syntactic but also semantic expectations. Unfortunately, we cannot directly prove this assumption with our study, as we did not detect the N400 effect (see possible reasons for this in the interpretation of the first hypothesis). However, we wanted to point out the difference between the masculine generic form and the gender-balanced form in the processing of the feminine verb. This is an indication that this specific issue cannot be fully explained by considering syntactic violations alone and would therefore need to be investigated in further studies.

The results largely reflect the discrepancy between the grammatical rules (especially the rule of masculine generics) and the agreement rules. When observing our gender-balanced forms, the agreement rules dictate that the last noun of the subject must agree with the following verb (e.g., in feminine-masculine word pair formation, the masculine form of the verb was expected to follow), as a closeness preference. However, in cases where there are different genders in the subject position (as in our case), grammar also prescribes the use of the verb in the masculine form, due to its unmarked use–the hierarchy rule (it is semantically “neutral” and has the greatest potential to influence gender agreement; Willer-Gold et al., 2016). Linguistic practice shows that the latter is also true regardless of whether the feminine form is directly adjacent to the verb (Toporišič, 2004; Marušič et al., 2015; Orešnik, 2015). In the cases where role nouns were presented with only one term, they were written in the masculine generic form, indicating masculine continuation. Thus, from the perspective of agreement rules, the results where the feminine verb was understood as a syntactic violation are to be expected. If we were to reverse the order of masculine and feminine forms in the gender-balanced form (putting the masculine form first and the feminine form second), the likelihood that the feminine forms would be recognized as appropriate would likely increase, but not drastically (see Willer-Gold et al., 2016). However, due to the rule of closeness, the masculine forms might be considered as less appropriate continuations in this scenario (although this is not very likely according to Willer-Gold et al., 2016). Thus, the order of the sentence structure, especially which gender form is presented last, may have a crucial influence on the processing of the antecedent and a different order could lead to different results.

However, we should also take into account the fact that the grammatical rules in Slovenian suggest that when we refer to a mixed-gender group of people (using the masculine generic form and the gender-balanced form), both the feminine and masculine continuations are considered as correct. The masculine generic form plays the role of a neutral and gender unmarked form that should be open to both feminine and masculine continuations, and the gender-balanced form is composed of both feminine and masculine forms and explicitly signals that we are addressing both women and men. Nevertheless, we found that participants interpreted only the masculine verb as a meaningful continuation of the antecedent in both the masculine form and the gender-balanced form, whereas feminine verb was perceived as a violation.

From our results, we conclude that in Slovenian the masculine grammatical gender and the rules of gender agreement, which emphasize the appropriateness of the masculine gender in almost every situation, strongly influence our perception of the congruence between antecedent and anaphora. It is also clear that participants rely heavily on their language habits when reading sentences that involve the resolution of an anaphora. Therefore, the masculine gender is considered the obvious choice, regardless of the form of the role name–and despite the fact that the feminine continuation would also be grammatically correct. Feminine forms (in nouns as word pairs or in verbs) therefore rarely turn out to be appropriate or even necessary in language use. However, our study has shown that gender-balanced forms are more open to the use of feminine verbs (feminine verbs elicited lower P600 when followed by a gender-balanced form than the masculine generic form), and we believe this is due to the explicit mention of the feminine gender in role name. Thus, we believe that new (and more gender-neutral) language practices offer a greater chance that women will be recognized as part of a gender-mixed group, and that feminine verbs will be considered more appropriate after feminine role nouns are mentioned in a word pair. The conclusion that gender-balanced forms are more open to feminine continuations than masculine generic forms is, in our opinion, very important and represents an important contribution to existing ERP research. Because of the interdisciplinary nature of the study, it has the potential to be interesting and useful to a variety of audiences, and it is the foundation on which we will build our future research.

### Study strengths and limitations

This is the first ERP study in Slovenian that has looked at the effects of processing grammatical gender in role names, and one of the first ERP studies in general to test the inclusivity of gender-balanced forms. Analyzes of Slovenian in these types of studies are welcome for several reasons. They have the potential to contribute to a better connection and understanding of Slavic languages by showing the similarities but also the differences between Slavic languages in the way they process gender information. The same applies to languages with grammatical gender as well as languages with natural gender, which could benefit from the use of Slovene in comparison to other languages (Spanish, German, Italian, English, etc.). The inclusion of Slovene in the group of languages studied with the ERP method could contribute to a better understanding of the specifics of processing gender information in general, but in particular to a better understanding of the effects that occur in language processing and to a clearer interpretation of these effects (referring to many open questions about the N400 and P600 effects in recent theories).

The great strength of this study is also that it was preregistered and that the majority of all procedures and analyzes were planned during preregistration.

A limitation of the study, which can also be considered a strength, is the use of an anaphoric verb instead of an anaphoric noun/pronoun used in the methodological frameworks of previous studies. In Slovenian, it was not possible to place violations in one word–noun (which contributes to a more explicit perception of violations as well as a clear connection between the anaphora and the antecedent) while maintaining the image of a gender mixed group and preserving a sense of naturalness in reading. Because of the placement of gender in the anaphoric verb, we expected that the connection between the measured verb and the role noun might seem unclear, especially because many other words were presented between role nouns at the beginning of the first sentence and the verb in the second sentence (nouns, adjectives, even verbs). This might have contributed to the fact that participants did not have a clear idea of what or to whom the anaphoric verb referred, and therefore perceived it as a (mainly syntactic) error. However, we should also acknowledge the innovation and novelty of this approach, which could have various implications for understanding language processing and should be further tested in other methodological frameworks and other languages.

In addition, the design of the experimental statements could have an important impact on the results for gender-balanced examples. Due to the need for consistency in the experimental sentences and the motivation that the sentences should sound as natural as possible, the gender-balanced forms always introduced the feminine noun first and the masculine noun second. This may have influenced the evaluation of relevance and agreement of the feminine/masculine verb. It would be interesting to investigate how the reverse order in which a word pair ends with a female noun would affect the processing of the feminine and/or masculine verb. If we were able to explore this aspect as well, it would give us a lot of valuable information about the influence of the closeness rule (and hierarchy) and provide a more solid basis for interpreting the current results, as well as the ability to compare the results of different sequences of role names. This is an important starting point for our further research.

We should also consider the possibility that although participants were unaware of the purpose of the study until it was completed, they were able to identify the purpose based on the sentence types presented and the task. This awareness could have influenced the results and had cultural implications for the conclusions. In this context, participants could also be motivated (due to the way the experiment works) to focus more on syntactic correctness and not consider the semantic dimension. However, changing the existing model for conducting these experiments was out of the question, as we did not want to lose the credibility of the results and the possibility of comparison with other studies.

The present study had some (already mentioned) limitations (limited number of conditions, anaphoric verb, gender-balanced role name sequences, etc.), but considering the fact that it is the first ERP study in Slovenian, we believe that it offers a lot of potential for further research involving other important factors affecting the processing of gendered referents. One of the necessary aspects to be included in this type of research is gender stereotypes or gender typicality of role names as an additional/independent condition and observing whether the acceptance of feminine forms in the verb would increase in this scenario, especially in the case of typically feminine occupations.

## Conclusion

In the present study, we investigated how grammatical gender affects the processing of referents in Slovenian. Specifically, we focused on how grammatically masculine forms (as generics) and the combination of different grammatical gender forms together (as gender-balanced word pairs) affect the processing of differently gendered referents. Although our initial focus was on exploring the effects of mental representations triggered by both forms, we found that Slovenian, as an extremely grammaticalized language, is highly conditioned and determined by grammatical and agreement rules. These rules largely overshadow the semantic associations (of women or men) that occur when reading a role name. The influence of grammatical rules (masculine generics, agreement rules) in grammatical gender languages has also been highlighted in some previous studies (e.g., Garnham et al., 2002; Irmen and Roßberg, 2004; Gygax et al., 2008), claiming that they are difficult to oppose and may even prevail against semantic information in the language.

We must also take into account the fact that the experimental material was formulated in such a way that both grammatical rules, the closeness rule and the rule of masculine generics, are more in line with the masculine continuation. Considering the fact that we did not find any ERP components indicating violations with the masculine verb, and that the ERP component that we found in the context of the feminine verb was P600, we can conclude that the rule of closeness and the generic rule had a decisive influence on the processing of gender information.

Another important finding is that gender-balanced form has been shown to be more open to feminine and masculine interpretations. We found that when we explicitly mention a woman in the role name (gender-balanced form), violations of feminine verbs are less pronounced (or more acceptable) than violations of feminine verbs that follow the masculine generic form (which includes women in meaning but not in form). This was evident when feminine verbs caused a positive shift at 600 ms presented after gender-balanced nouns, but the positivity was smaller than in the case of nouns in generic (masculine) form. We took this result as an indication of the need for further investigation of this phenomenon, which will give us a clearer understanding of the factors (syntactic only or semantic as well) involved in the processing of gender-balanced forms.

The results of this interdisciplinary study are aimed at different audiences: those interested in the processing of gender information, the effects of processing specific role names, etc. However, an important goal of this study is also to contribute to the debates in which language is seen as an important tool for the production and reproduction of sexism in society and in the labor market.

## Data availability statement

The data, materials, and analysis scripts can be found on Open Science Framework (OSF): https://dx.doi.org/10.17605/OSF.IO/MAZHU.

## Ethics statement

The studies involving human participants were reviewed and approved by University of Ljubljana, Faculty of Social Sciences, Kardeljeva ploščad 5, Ljubljana, Slovenia. The participants provided their written informed consent to participate in this study.

## Author contributions

JML was responsible for conceptualization, investigation, writing the original draft, and for funding acquisition. AM was responsible for the methodology, software, formal analysis, investigation, data curation, writing the original draft, and visualization. JB and AKM were responsible for writing—review and editing and supervision. All authors contributed to the article and approved the submitted version.

## Footnotes

1
https://dx.doi.org/10.17605/OSF.IO/MAZHU
