# Supplementary material for: The effects of grammatical gender on the processing of occupational role names in Slovene: An event-related potential study
Source: Front Psychol. 2022 Dec 15;13:1010708. doi: 10.3389/fpsyg.2022.1010708 (PMC9807137; doi:10.3389/fpsyg.2022.1010708)
Supplement: Supplementary file 1 [file Data_Sheet_1.doc]

Supplementary Material

Supplementary material for the article: The effects of grammatical gender on processing occupational role names in Slovene: an ERP study. Authors: Jasna Mikić Ljubi, Andraž Matkovič, Jurij Bon and Aleksandra Kanjuo Mrčela.

# Supplementary Figures and Tables

***Table S1.*** *Results of logistic regression for behavioral results (answers 'yes' vs. 'no').*

| comparison | term | *B* | *SE* | *z* | *p* | *p* (FDR) | < .05 |
| --- | --- | --- | --- | --- | --- | --- | --- |
| feminine verb vs. masculine | intercept | -0.94 | 0.28 | -3.36 | .001 | .002 | * |
|  | anaphora (masculine verb) | 3.44 | 0.12 | 28.51 | < .001 | < .001 | * |
|  | grammatical form (generic) | -0.17 | 0.10 | -1.67 | .095 | .146 |  |
|  | interaction | 0.04 | 0.16 | 0.24 | .811 | .811 |  |
|  | SD (intercept) | 1.38 |  |  |  |  |  |
| feminine verb vs. semantic error | intercept | -1.14 | 0.33 | -3.40 | .001 | .002 | * |
|  | anaphora (semantic error) | -2.82 | 0.21 | -13.55 | < .001 | < .001 | * |
|  | grammatical form (generic) | -0.19 | 0.11 | -1.75 | .080 | .134 |  |
|  | interaction | -0.31 | 0.30 | -1.03 | .304 | .338 |  |
|  | SD (intercept) | 1.65 |  |  |  |  |  |
| feminine verb vs. syntactic error | intercept | -1.15 | 0.42 | -2.75 | .006 | .012 | * |
|  | anaphora (syntactic error) | -0.77 | 0.16 | -4.89 | < .001 | < .001 | * |
|  | grammatical form (generic) | -0.23 | 0.12 | -1.92 | .055 | .100 |  |
|  | interaction | 0.28 | 0.22 | 1.28 | .202 | .289 |  |
|  | SD (intercept) | 2.09 |  |  |  |  |  |
| masculine verb vs. semantic error | intercept | 2.29 | 0.19 | 11.97 | < .001 | < .001 | * |
|  | anaphora (semantic error) | -5.32 | 0.20 | -26.01 | < .001 | < .001 | * |
|  | grammatical form (generic) | -0.14 | 0.13 | -1.13 | .258 | .331 |  |
|  | interaction | -0.31 | 0.29 | -1.04 | .299 | .338 |  |
|  | SD (intercept) | 0.84 |  |  |  |  |  |
| masculine verb vs. syntactic error | intercept | 2.43 | 0.26 | 9.44 | < .001 | < .001 | * |
|  | anaphora (syntactic error) | -3.89 | 0.15 | -25.62 | < .001 | < .001 | * |
|  | grammatical form (generic) | -0.14 | 0.12 | -1.12 | .265 | .331 |  |
|  | interaction | 0.17 | 0.20 | 0.88 | .378 | .398 |  |
|  | SD (intercept) | 1.22 |  |  |  |  |  |


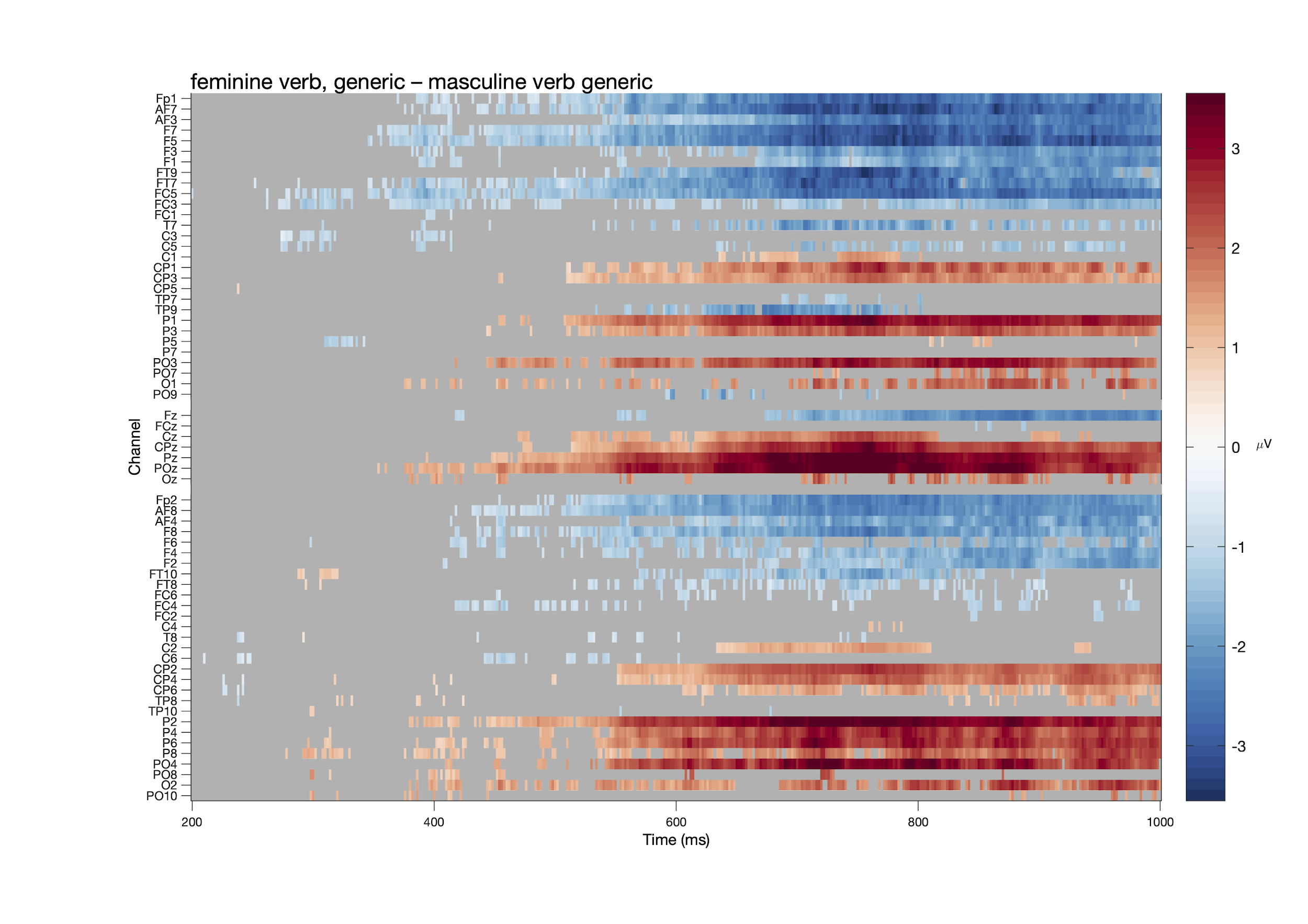


**Figure S1.** Mass univariate tests for the difference between feminine verb, generic, and masculine verb, generic, conditions. Non-significant differences are colored gray. There are large and widespread differences between conditions from 500 to 1000 ms with feminine verb, generic, condition having higher activity in right parieto-occipital region and smaller activity in left frontal region. The results indicate a large P600 in the feminine verb, generic, condition and the absence of a P600 in the masculine verb, generic, condition.

*
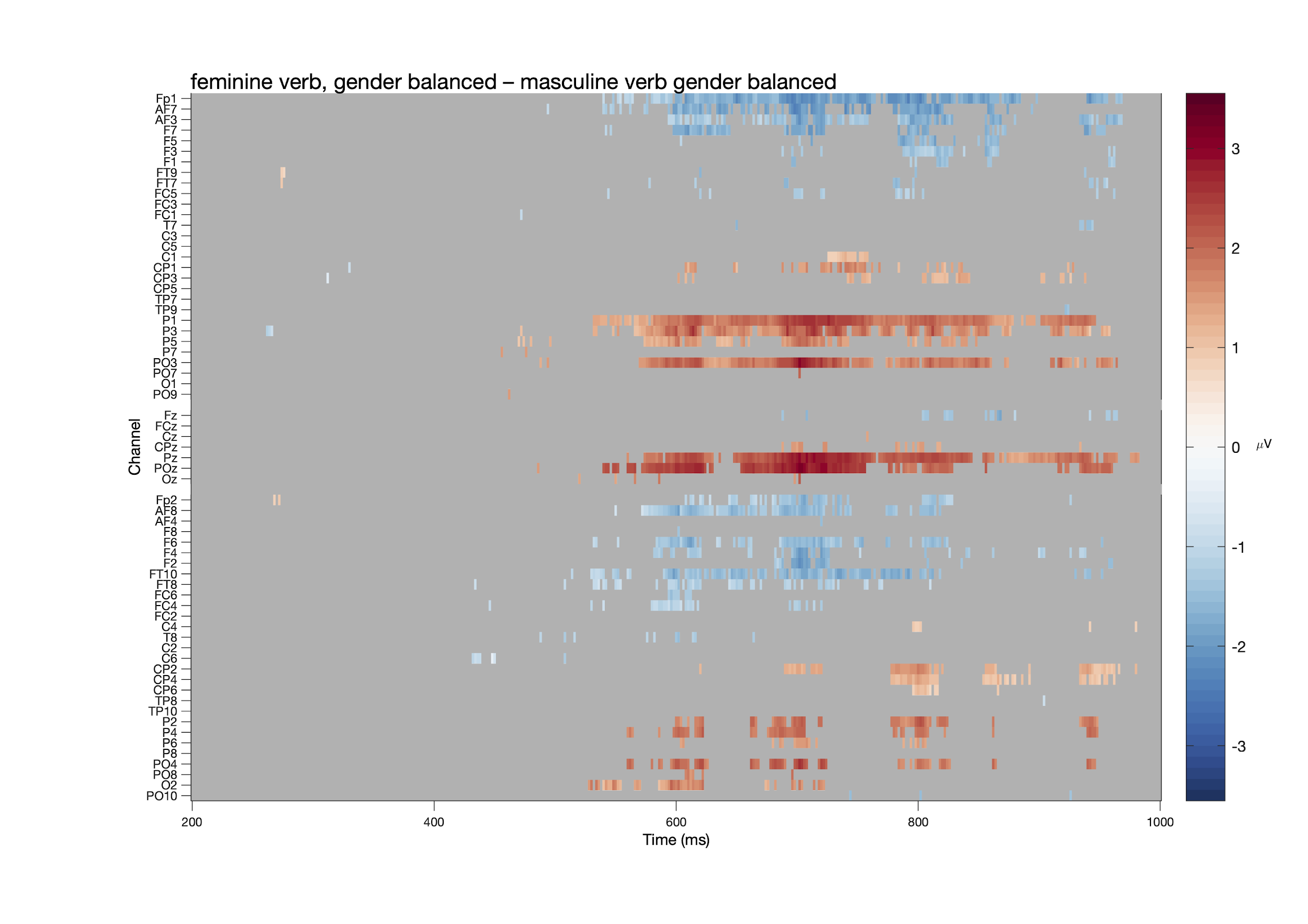
*

**Figure S2.** Mass univariate tests for the difference between feminine verb, gender-balanced, and masculine verb, gender-balanced, conditions. Non-significant differences are colored gray. There are smaller, localized, differences between conditions. In the feminine verb, gender-balanced, condition activity in was greater in the right parieto-occipital area from 550 to 950 ms and smaller activity in the frontal channels. The results indicate P600 in feminine verb, gender-balanced, condition and the absence of P600 in masculine verb, gender-balanced, condition.


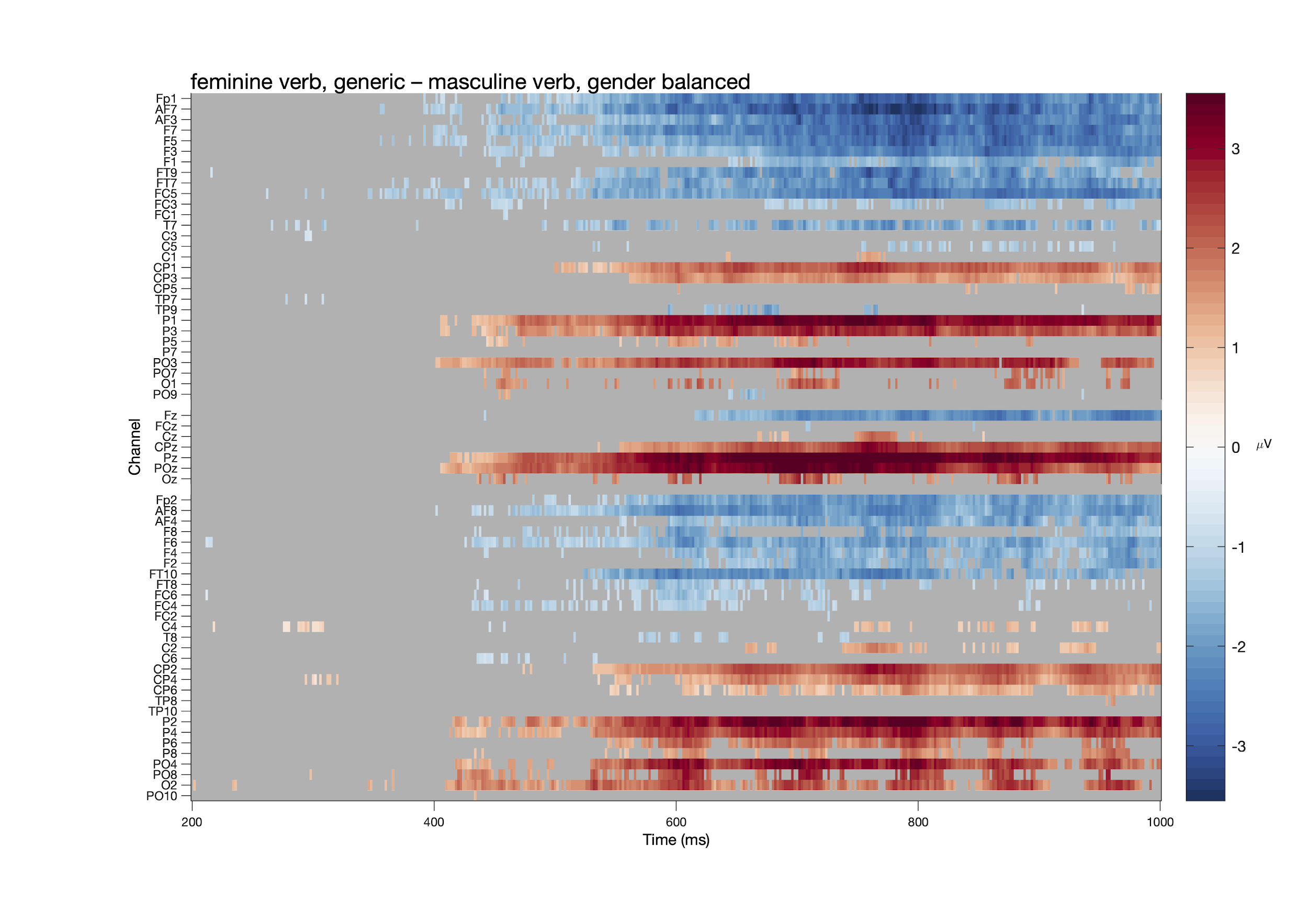


**Figure S3.** Mass univariate tests for the difference between the feminine verb, generic, and the masculine verb, gender-balanced, conditions. Non-significant differences are colored gray. There are large and widespread differences between conditions from 400 to 1000 ms, with the feminine verb, generic, condition having higher activity in the right parieto-occipital area and smaller activity in the left frontal area. The results indicate a large P600 in feminine verb, generic, condition and the absence of a P600 in the masculine verb, gender-balanced, condition.


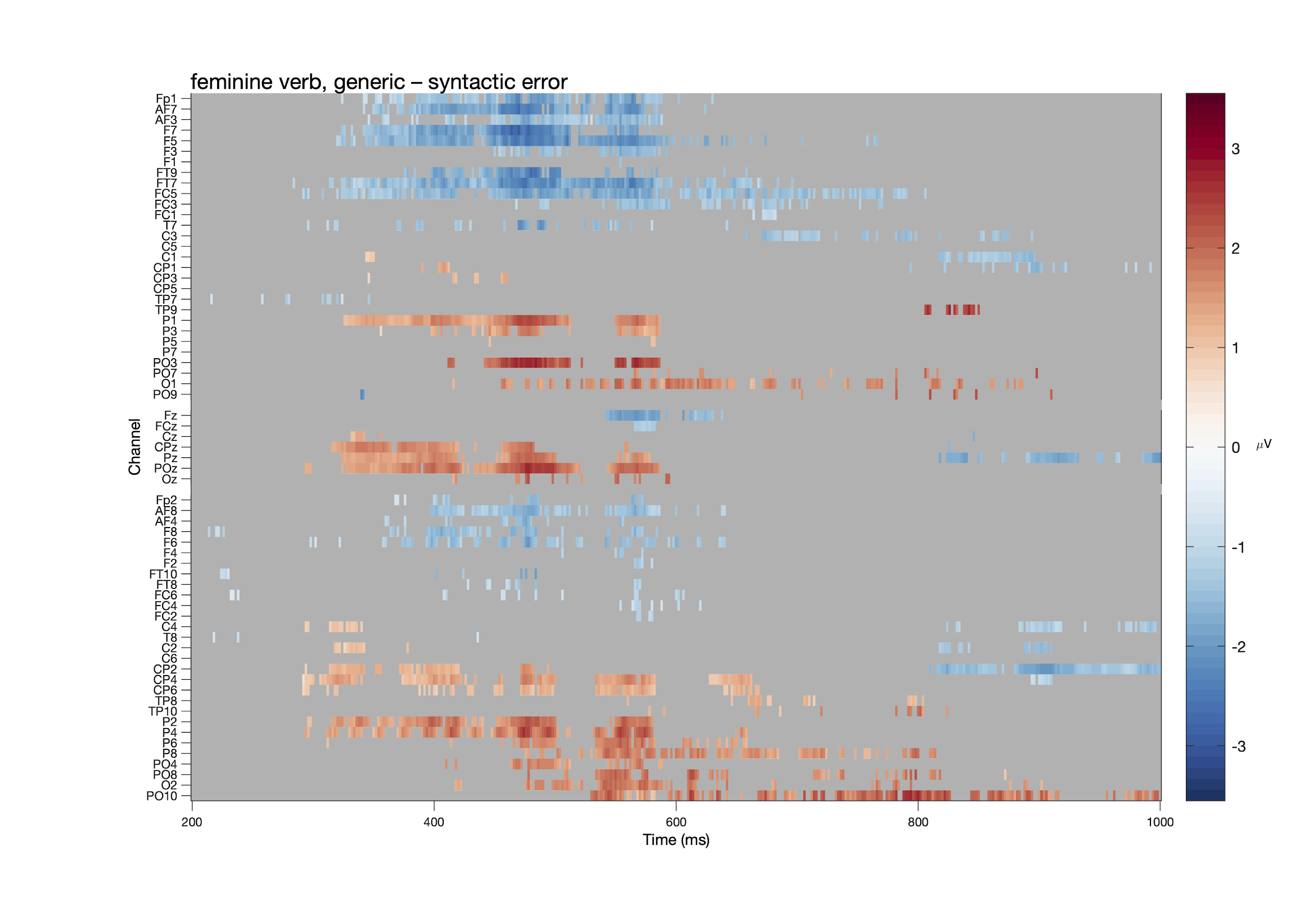


**Figure S4.** Mass univariate tests for the difference between feminine verb, generic, and syntactic error conditions. Non-significant differences are colored gray. There was larger negativity in the syntactic error condition from 300 to 600 ms in the frontal channels and smaller positivity in the parietal channels, indicating larger N400 amplitude in the syntactic error condition.


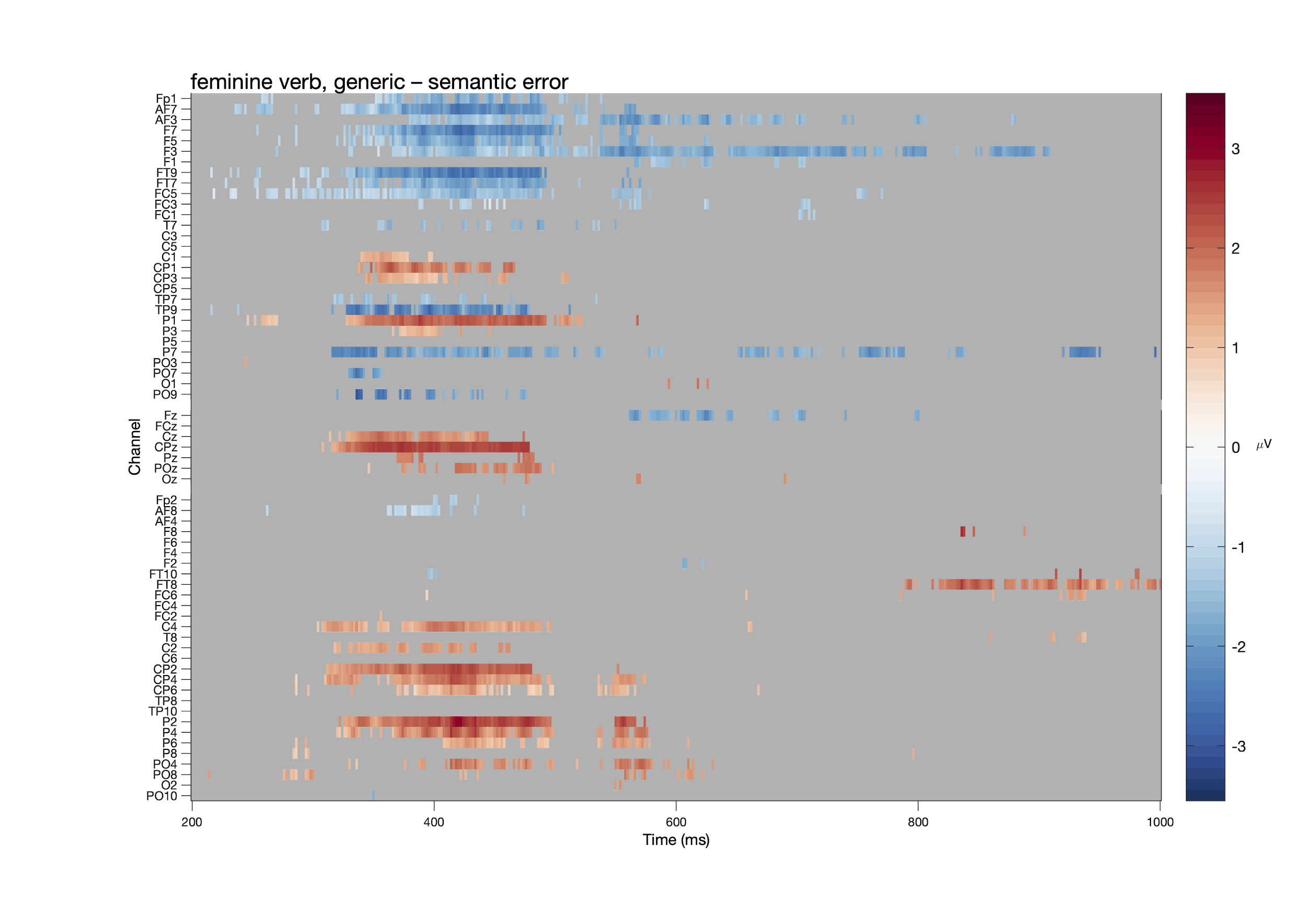


**Figure S5.** Mass univariate tests for the difference between feminine verb, generic, and semantic error conditions. Non-significant differences are colored gray. There was a larger negativity in the semantic error condition from 300 to 600 ms in the frontal channels and smaller positivity in the parietal channels, indicating larger N400 amplitude in the semantic error condition.


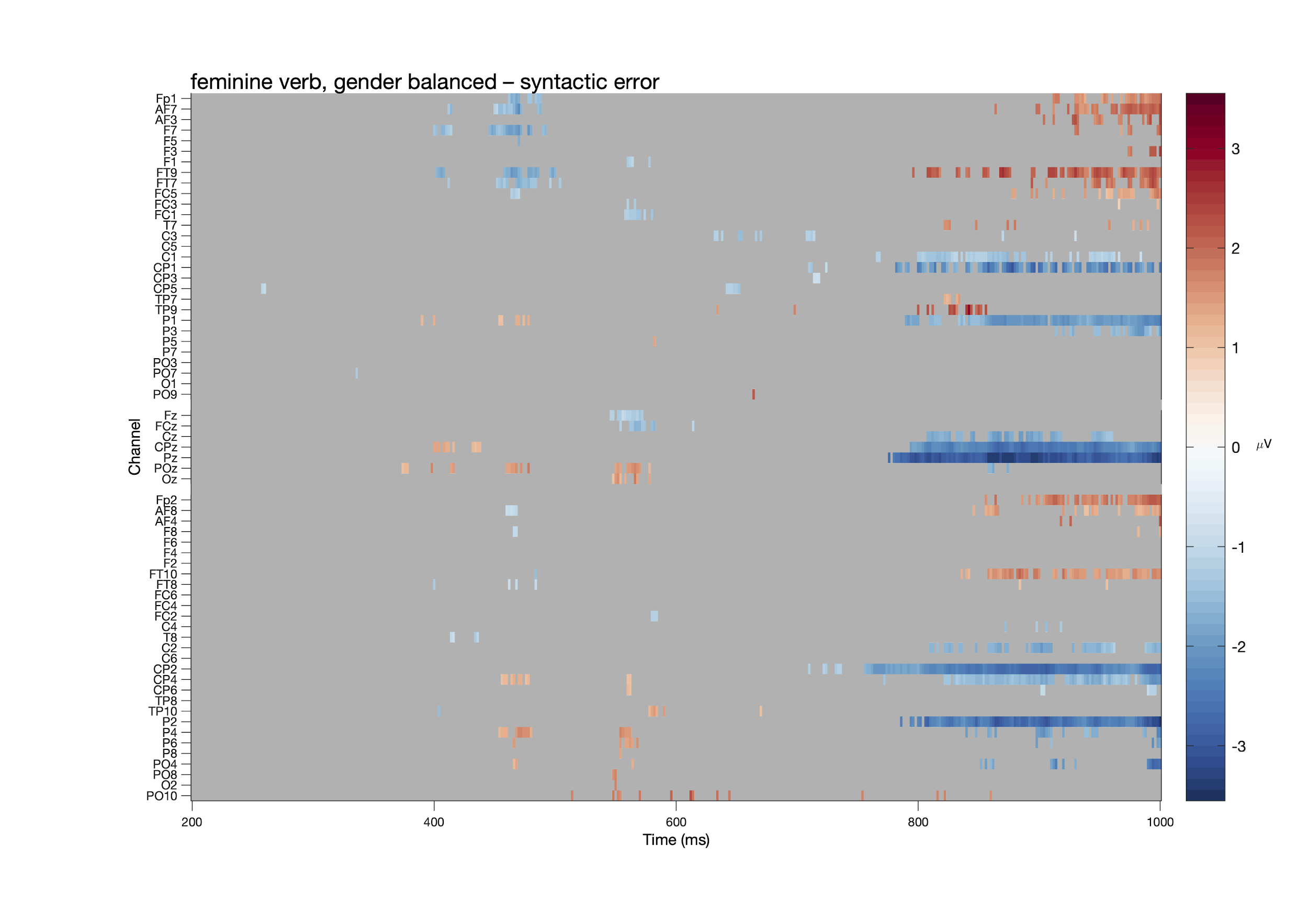


**Figure S6.** Mass univariate tests for the difference between feminine verb, gender-balanced, and syntactic error conditions. Non-significant differences are colored gray. In the syntactic erroc condition, activity was larger in parietal channels from 800 to 1000 ms. Given that P600 was present in both conditions (Figure 3 in the main text), the results suggest longer P600 activation in the syntactic error condition. Differences around 450–500 ms indicate a slightly larger N400 amplitude for the syntactic error condition.


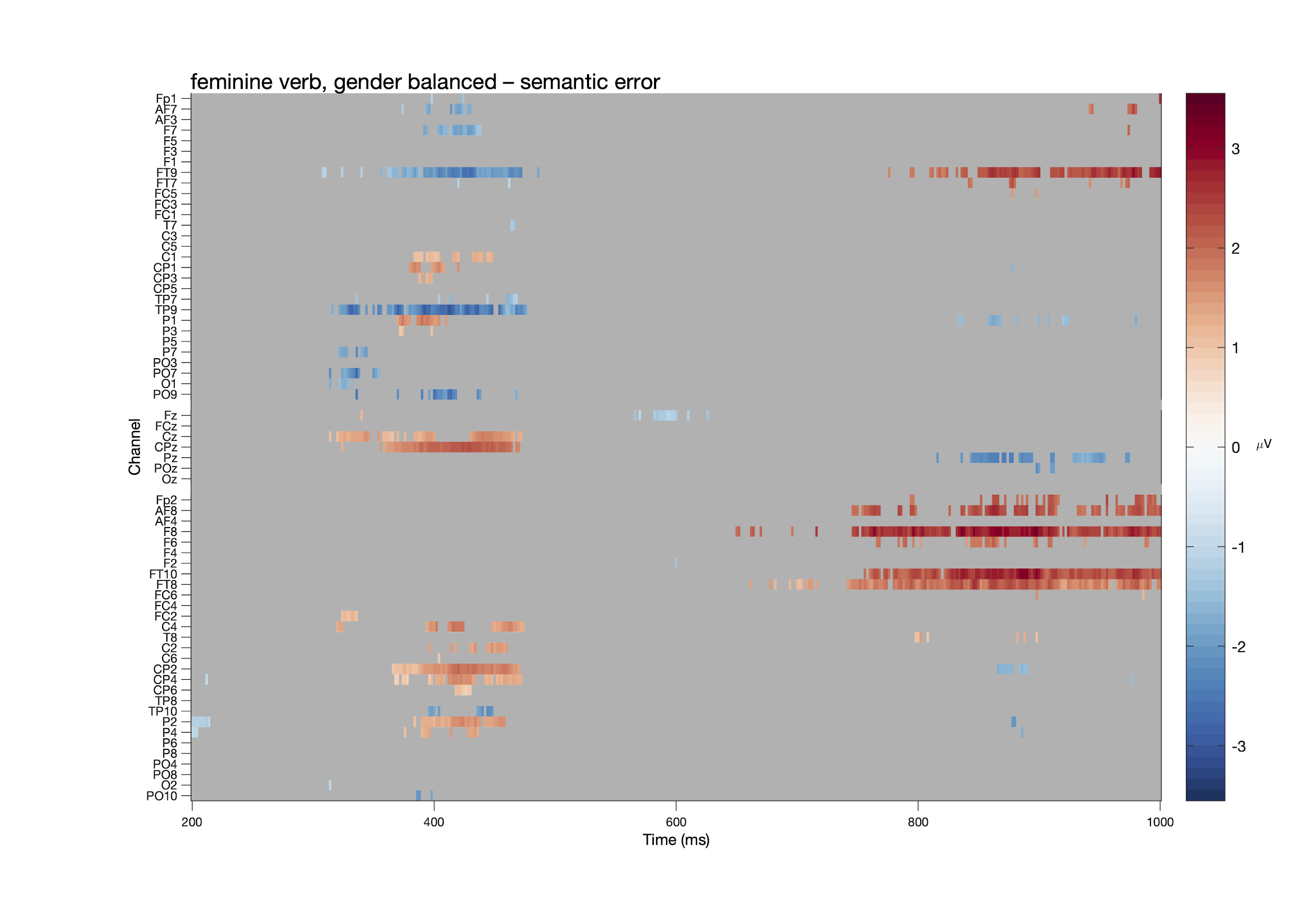


**Figure S7.** Mass univariate tests for the difference between feminine verb, gender-balanced, and semantic error conditions. Non-significant differences are colored gray. In the feminine verb, gender-balanced condition, activity was larger in the frontal channels from 800 to 1000 ms. Small differences were also present around 400 ms. The results indicate longer P600 activation and larger N400 in the semantic error condition.

# Literal English glosses for examples of experimental sentences in the manuscript

(1)  Generic masculine form in noun + feminine verb:

| Kirurgi | so | ponovno | delali | nadure. |
| --- | --- | --- | --- | --- |
| surgeons  _masculine form_ | auxiliary verb *to be* for the past tense | again | *to work* in past tense | overtime. |
| Večini | je bilo | odveč, | saj | bi |
| For most | was / to / it | unnecessary | as | would |
| svoj | čas | raje | *preživljale* | s |
| their | time | rather | *to spend* with feminine suffix in plural past tense | with |
| partnerjem. |  |  |  |  |
| partner. |  |  |  |  |

(2)  Generic masculine form in noun + masculine verb:

| Telefonisti | so | si | med | pavzo |
| --- | --- | --- | --- | --- |
| Telephonists _masculine form_ | auxiliary verb *to be* for the past tense | reflexive personal pronoun | during | break |
| privoščili | kavo. | Ker | je bila | nekaterim |
| to indulge in past tense | coffee. | Because | (it) was | to some |
| pregrenka | so | jo | *pili* | z |
| too bitter | auxiliary verb *to be* for the past tense | it | *to drink* verb with masculine suffix in pl. in past tense | with |
| mlekom. |  |  |  |  |
| milk. |  |  |  |  |

(3)  Gender balanced form + feminine verb:

| Skladateljice in skladatelji | so | se | navadili | samostojnosti |
| --- | --- | --- | --- | --- |
| Composers _feminine form_ and composers _masculine  form_ | auxiliary verb *to be* for the past tense | reflexive personal pronoun | accustomed to | automony |
| pri | svojem | delu. | Mnogim | se je |
| at | their | work. | To many | it has |
| zato | zgodilo, | da | so | se |
| therefore | happened | that | auxiliary verb *to be* for the past tense | reflexive personal pronoun |
| skupinskega | dela | popolnoma | *odvadile.* |  |
| group | work | completely | *to be disengaged* verb with feminine suffix in pl. in past tense. |  |

(4)  Gender balanced form + masculine verb:

| Čistilke in čistilci | so | imeli | pozno | malico. |
| --- | --- | --- | --- | --- |
| Cleaners _feminine form_ and cleaners _masculine  form_ | auxiliary verb *to be* for the past tense | to have | late | lunch. |
| Mnogim | je | to | ustrezalo, | saj |
| To many | is | that | suited | because |
| so | si | lahko | *vzeli* | več |
| auxiliary verb *to be* for the past tense | reflexive personal pronoun | could | *to take* verb with masculine suffix in pl. in past tense | more |
| časa | zanjo. |  |  |  |
| time | for it. |  |  |  |
